# Linear and Nonlinear Optical Properties from TDOMP2 Theory

**DOI:** 10.1021/acs.jctc.1c01309

**Published:** 2022-04-18

**Authors:** Håkon Emil Kristiansen, Benedicte Sverdrup Ofstad, Eirill Hauge, Einar Aurbakken, Øyvind Sigmundson Schøyen, Simen Kvaal, Thomas Bondo Pedersen

**Affiliations:** † Hylleraas Centre for Quantum Molecular Sciences, Department of Chemistry, University of Oslo, Oslo N-0315, Norway; ‡ Simula Research Laboratory, Kristian Augusts Gate 23, Oslo 0164, Norway; § Department of Physics, University of Oslo, Oslo N-0316, Norway; ∥ Centre for Advanced Study at the Norwegian Academy of Science and Letters, Drammensveien 78, Oslo N-0271, Norway

## Abstract

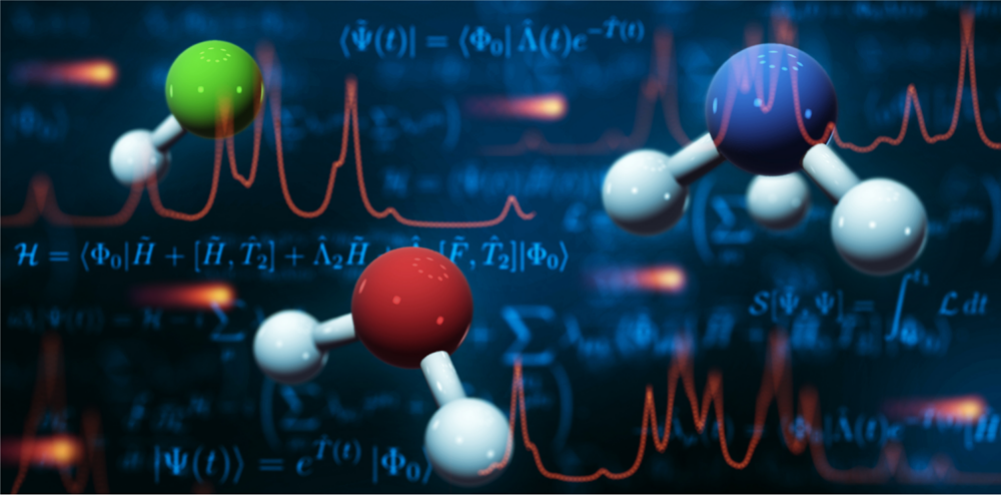

We present a derivation of real-time (RT) time-dependent orbital-optimized Møller–Plesset (TDOMP2) theory and its biorthogonal companion, time-dependent non-orthogonal OMP2 theory, starting from the time-dependent bivariational principle and a parametrization based on the exponential orbital-rotation operator formulation commonly used in the time-independent molecular electronic structure theory. We apply the TDOMP2 method to extract absorption spectra and frequency-dependent polarizabilities and first hyperpolarizabilities from RT simulations, comparing the results with those obtained from conventional time-dependent coupled-cluster singles and doubles (TDCCSD) simulations and from its second-order approximation, TDCC2. We also compare our results with those from CCSD and CC2 linear and quadratic response theories. Our results indicate that while TDOMP2 absorption spectra are of the same quality as TDCC2 spectra, including core excitations where optimized orbitals might be particularly important, frequency-dependent polarizabilities and hyperpolarizabilities from TDOMP2 simulations are significantly closer to TDCCSD results than those from TDCC2 simulations.

## 1 Introduction

The correct semiclassical description of interactions between matter and temporally oscillating electromagnetic fields must start from time-dependent quantum mechanics. Historically, the most often used approach within molecular electronic structure theory has been time-dependent perturbation theory, where the time-dependent Schrödinger equation is solved order by order in the external field strength, leading to a response theory of molecular properties in the frequency domain through the application of a series of Fourier transforms.^1^ Response theory has the advantage that it directly addresses the quantities that are used for the interpretation of experimental measurements, such as one- and two-photon transition moments and frequency-dependent electric-dipole polarizabilities and hyperpolarizabilities, which may be expressed in terms of transition energies and stationary-state wave functions that can, at least in principle, be obtained from the time-independent Schrödinger equation for the particle system alone. A major disadvantage is that time resolution is lost when going from the time domain to the frequency domain. The obvious solution would be to skip the Fourier transforms and instead work directly in the time domain. This, however, implies that the time-dependent Schrödinger equation must be solved order by order in a discretized time series, making the approach much too computationally demanding for higher-order properties. Instead, so-called *real-time* (RT) methods have received increasing attention in recent years—see, for example, the review of RT time-dependent electronic structure theory by Li et al.^2^

RT methods approximate the solution of the time-dependent Schrödinger equation without perturbation expansions and, thus, contain information about the response of the atomic or molecular electrons to external electromagnetic fields to all orders in perturbation theory. Even extremely nonlinear processes that are practically out of reach within response theory, such as high harmonic generation and time-resolved one- and many-electron ionization probability amplitudes, are accessible with RT methods, see ref (2) and references therein. Moreover, because RT methods include the field explicitly in the simulation, it becomes possible to investigate the detailed dependence on laser parameters such as intensity, frequency distribution, pulse shape, and delay between pump and probe pulses without making explicit assumptions about the perturbation order of the electronic processes involved.

Although RT methods are usually much simpler to implement than response theory (typically, the same code is needed as for ground-state calculations, only generalized to complex parameters), a major downside of RT methods is the increased computational cost arising from the discretization of time. Thousands or even hundreds of thousands of time steps are needed, each associated with a cost comparable to one (or a few) iterations of a ground-state optimization with the same (time-independent) method. In addition, the basis set requirements are generally more demanding because, in principle, all the excited states and even continuum states may be involved in the dynamics, and acceleration techniques commonly used for the ground-state and response calculations may not be generally applicable for RT simulations with all possible external electromagnetic fields.

It is no surprise, therefore, that the most widely used RT electronic structure method is RT time-dependent density-functional theory (RT-TDDFT).^2−5^ Highly accurate wave function-based RT methods have also been developed, including multiconfigurational time-dependent Hartree–Fock (MCTDHF)^6−9^ theory and related complete, restricted, and generalized active space formulations.^10−12^ Avoiding the factorial computational scaling caused by the full configuration interaction (FCI) treatment at the heart of these approaches, time-dependent extensions of single-reference coupled-cluster (CC) theory^13^ and equation-of-motion CC (EOM-CC) theory^14,15^ have been increasingly often used to simulate laser-driven many-electron dynamics in the time domain in recent years.^16−33^ The two approaches, time-dependent CC (TDCC) and time-dependent EOM-CC (TD-EOM-CC) theories, differ in their parametrization of the time-dependent left and right wave functions. While TDCC theory propagates the well-known exponential *Ansätze* for the wave functions, TD-EOM-CC theory expresses them as the linear combinations of EOM-CC left and right eigenstates. While both approaches are expected to give similar results (and, indeed, appear to do so, see ref (33)) for weak-field processes, only the TDCC theory (albeit with dynamical orbitals) has been successfully applied to strong-field phenomena such as ionization dynamics and high harmonic generation^20^ to date.

Although the original formulation of TDCC theory in nuclear physics was based on time-dependent Hartree–Fock (HF) orbitals,^34^ conventional TDCC theory is formulated with a static reference determinant, the HF ground state, which is kept fixed during the dynamics in agreement with the conventional formulation of CC response (LRCC) theory.^35,36^ The fixed orbital space has some unwanted side effects, however. Gauge invariance is lost in truncated TDCC theory (but recovered in the FCI limit),^37,38^ severe numerical challenges arise as the CC ground state is depleted during the dynamics (e.g., in ground-excited state Rabi oscillations),^21,29^ and it becomes impossible to reduce the computational effort while maintaining accuracy by splitting the orbital space into active and inactive orbitals for the correlated treatment, as required to efficiently describe ionization dynamics.^17^ These deficiencies can, at least partially, be circumvented by allowing the orbitals to move in concert with the electron correlation. In practice, this is done by replacing the single excitations (and de-excitations) of conventional CC theory with full orbital rotations. This, in turn, can be done in two ways. Within orbital-optimized CC (OCC) theory,^37,39,40^ the orbitals are required to remain orthonormal, whereas within nonorthogonal OCC (NOCC) theory^17,38^ they are only required to be biorthonormal. The orthonormality constraint has an unfortunate side effect in the sense that the OCC theory does not converge to the FCI solution in the limit of full rank cluster operators for three or more electrons, as pointed out by Köhn and Olsen.^41^ On the other hand, Myhre^42^ recently showed that the NOCC theory may converge to the correct FCI limit for any number of electrons. In practice, however, time-dependent OCC (TDOCC) theory does not appear to deviate from the FCI limit by any significant amount.^20^

The computational scaling with respect to the size of the basis set and with respect to the number of electrons in TDOCC and time-dependent NOCC (TDNOCC) theory is essentially identical to that of conventional TDCC theory with identical truncation of the cluster operators. The lowest-level truncation, after double excitations, yields the TDOCCD and TDNOCCD methods that both scale as , which is significantly more expensive than the formal  scaling of RT-TDDFT. In order to bring down the computational cost to a more tractable level, Pathak et al.^26,27^ generalized the orbital-optimized second-order Møller–Plesset (OMP2)^43^ method to the time domain and demonstrated that the resulting TDOMP2 method provides a reasonably accurate and gauge invariant description of highly nonlinear optical processes.

In this work, we assess the description of linear and quadratic optical properties within the TDOMP2 approximation. First, we review the TDCC theory and its second-order approximation TDCC2. Second, we review TDCC theories with dynamic orbitals—TDNOCC and TDOCC theory—as obtained from the time-dependent bivariational principle, and introduce the second-order approximations TDNOMP2 and TDOMP2. Finally, we compute linear (one-photon) absorption spectra and frequency-dependent polarizabilities and first hyperpolarizabilities with the TDOMP2, TDCCSD, and TDCC2 methods and compare them with results from CC2 and CCSD linear and quadratic response theory.

## 2 Theory

### 2.1 Notation

We consider a system of *N* interacting electrons described by the second-quantized Hamiltonian
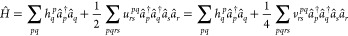
1where *â*_*p*_^†^ (*â*_*p*_) are creation (annihilation) operators associated with a finite set of L orthonormal spin orbitals {ϕ_*p*_}_*p*=1_^L^. The one- and two-body matrix elements h_q_^p^ and u_rs_^pq^, respectively, are defined as
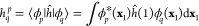
2
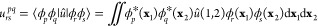
3where **x**_*i*_ = (**r**_*i*_, σ_*i*_) refers to the combined spatial-spin coordinate of electron *i*. The anti-symmetrized two-body matrix elements v_rs_^pq^ are given by
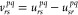
4

### 2.2 TDCC2 Approximation

The TDCC *Ansätze* for the left and right CC wave functions are defined by
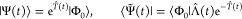
5where |Φ_0_⟩ is a reference determinant built from orthonormal spin orbitals, typically taken as the HF ground-state determinant. The chosen reference determinant splits the orbital set into occupied orbitals denoted by subscripts *i*, *j*, *k*, *l* and virtual orbitals denoted by subscripts *a*, *b*, *c*, *d*. Subscripts *p*, *q*, *r*, *s* are used to denote general orbitals. The cluster operators *T̂*(*t*) and Λ̂(*t*) are given by
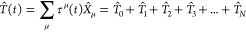
6
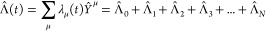
7where μ denotes the excitations of rank 0, 1, 2, 3, ..., *N*, and the excitation and de-excitation operators *X̂*_μ_ and *Ŷ*^μ^, respectively, are defined by
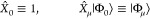
8
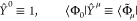
9such that ⟨Φ̃_μ_|Φ_ν_⟩ = δ_μν_. The rank-0 cluster operators are included to describe the phase and (intermediate) normalization of the CC state.^21^

The equations of motion for the wave function parameters are obtained from the bivariational action functional used by Arponen^44^
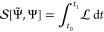
10where the CC Lagrangian is given by
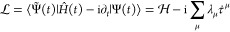
11and the Hamilton function  is given by
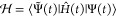
12

The requirement that  be stationary with respect to variations of the complex parameters *z*_μ_ ∈ {τ^μ^, λ_μ_} leads to the Euler–Lagrange equations
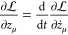
13

Taking the required derivatives yields the equations of motion for the amplitudes,
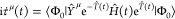
14
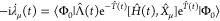
15

Note that λ_0_(*t*) is a constant, which we choose such that the intermediate normalization condition ⟨Ψ̃(*t*)|Ψ(*t*)⟩ = 1 is satisfied, whereas the phase amplitude τ_0_ generally depends nontrivially on time.^21^ The phase amplitude may, however, be ignored as long as we are only interested in the time evolution of expectation values.^35^ For other quantities, such as the autocorrelation of the CC state^21^ or certain stationary-state populations,^30^ the phase amplitude is needed. In the present work, we will only consider expectation values.

Truncation of the cluster operators after single and double excitations defines the TDCCSD method, which has an asymptotic scaling of . Defined as a second-order approximation to the TDCCSD method within many-body perturbation theory, the TDCC2 method^45^ reduces the asymptotic scaling to . In order to derive the TDCC2 equations, we partition the time-dependent Hamiltonian
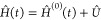
16into a zeroth-order term, *Ĥ*^(0)^(*t*) = *f̂* + *V̂*(*t*), where *f̂* is the Fock operator, and
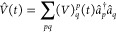
17is a time-dependent one-electron operator representing the interaction with an external field. The first-order term (the fluctuation potential) is defined as,
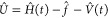
18

In the many-body perturbation analysis of the TDCCSD equations, the singles and doubles amplitudes are considered zeroth-order and first-order quantities, respectively. For notational convenience, the time dependence of the amplitudes and operators will be understood implicitly in the following.

The equations of motion are obtained by making the action given by eq 10 stationary with respect to variations of the amplitudes. The TDCC2 Lagrangian is obtained from the TDCCSD Lagrangian by retaining terms up to quadratic in the doubles amplitudes and the fluctuation potential
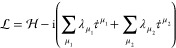
19

Introducing *T̂*_1_-transformed operators as
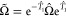
20the TDCC2 approximation to the TDCCSD Hamilton function becomes
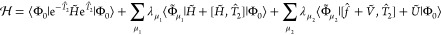
21

Note that the Fock operator appearing in the commutator in the last term is *notT̂*_1_ transformed. The Euler–Lagrange equations then yield equations of motion for the singles amplitudes
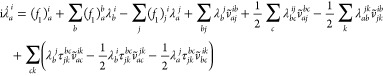
22
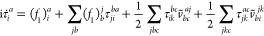
23and for the doubles amplitudes,
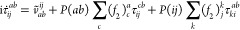
24
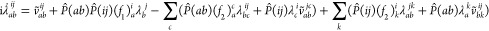
25Here, we have defined the fully and partially *T*_1_-transformed Fock matrices
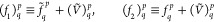
26and the operator *P̂*(*pq*) is an anti-symmetrizer defined by its action on the elements of an arbitrary tensor *M*: *P̂*(*pq*)*M*_*pq*_ = *M*_*pq*_ – *M*_*qp*_.

The presence of the untransformed Fock operator in eq 21 has a number of simplifying consequences. For example, the ground-state doubles amplitudes become explicit functions of the singles amplitudes, and the double excitation block of the EOM-CC Hamiltonian matrix (the CC Jacobian) becomes diagonal. In TDCC2 theory, however, it implies that the doubles amplitudes are not fully adjusted to the approximate orbital relaxation captured by the (zeroth-order) singles amplitudes. In order to test the consequences of this, we have implemented the TDCC2-b method of Kats et al.,^46^ where the fully *T*_1_-transformed Fock operator is used in eq 21.

### 2.3 Review of Time-dependent Coupled-Cluster Theories with Dynamic Orbitals

The TDOCC and TDNOCC *Ansätze* replace the singles amplitudes of conventional TDCC theory with unitary and non-unitary orbital rotations, respectively. For both types of orbital rotations, the left and right CC wave functions can be written on the form
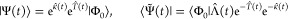
27where |Φ_0_⟩ is a static reference determinant built from orthonormal spin orbitals, typically taken as the HF ground-state determinant in analogy with conventional TDCC theory. The terminology of occupied and virtual orbitals thus refers to this reference determinant, although both subsets are changed by the time-dependent orbital rotations. Excluding singles amplitudes, the cluster operators *T̂*(*t*) and Λ̂(*t*) are given by
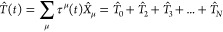
28
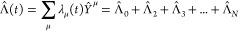
29where μ denotes excitations of ranks 0, 2, 3, ..., *N*, and the excitation and de-excitation operators *X̂*_μ_ and *Ŷ*^μ^ are defined the same way as in conventional TDCC theory [eqs 8 and 9], respectively. The exclusion of singles amplitudes is rigorously justified, as they become redundant when the orbitals are properly relaxed by the orbital-rotation operator exp(κ̂).^17,37,38^

In TDNOCC theory, the orbital rotations are non-unitary, that is, κ̂^†^ ≠ −κ̂. If κ̂ is restricted to be anti-Hermitian, we obtain TDOCC theory where the orbital rotations are unitary. However, this leads to the parametrization formally not converging to the FCI limit (for *N* > 2), as pointed out by Köhn and Olsen.^41^ On the other hand, Myhre^42^ showed that the proper FCI limit may be restored by non-unitary orbital rotations. Furthermore, it can be shown that occupied–occupied and virtual–virtual rotations are redundant,^17,37^ and that it is sufficient to consider κ̂(*t*) on the form
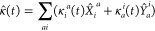
30

Using the Baker–Campbell–Hausdorff expansion, one can show that the similarity transforms of the creation and annihilation operators with exp(κ̂) are given by
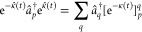
31
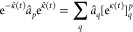
32

Recalling that explicit time dependence only appears in the interaction operator and in the wave function parameters, we will suppress the dependence on time in the notation. For a general one- and two-body operator Ω̂, the TDNOCC and TDOCC expectation value functionals can be written as
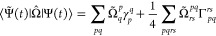
33where
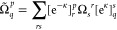
34
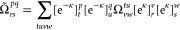
35and γ, Γ are the effective one- and two-body density matrices, respectively, given by
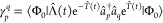
36
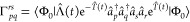
37

The equations of motion for the wave function parameters are, again, obtained from the Euler–Lagrange eq 13 for the full parameter set *z*_μ_ ∈ {τ^μ^, λ_μ_, κ_i_^a^, κ_a_^i^} with the Lagrangian given by
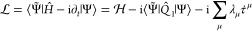
38where the Hamiltonian is given by eq 1. Here, the interaction with the external field (17) is absorbed into the one-body part of the Hamiltonian such that
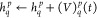
39

The operator *Q̂*_1_ is defined as
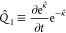
40and .

The detailed derivation of the equations of motion is greatly simplified by absorbing the orbital rotation in the Hamiltonian at each point in time, *Ĥ* ← exp(−κ̂)*Ĥ* exp(κ̂), which amounts to temporally local updates of the Hamiltonian integrals according to eqs 34 and 35. This allows us to compute the temporally local derivatives of the Lagrangian with respect to the parameters at the point κ̂ = 0, such that, for example, the rather complicated operator *Q̂*_1_ becomes the much simpler operator . We thus find that the equations of motion for the cluster amplitudes are given by
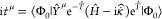
41
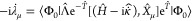
42where the right-hand sides are essentially identical to the usual amplitude equations of CC theory, with additional terms arising from the one-body operator . As in conventional TDCC theory, λ_0_ is constant and may be chosen such that intermediate normalization is preserved.^21^ In the same manner, we may derive the equations of motion for the orbital-rotation parameters as
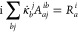
43
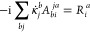
44where the right-hand sides are given by eqs 30a and 30b in ref (17), and
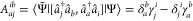
45

Equations 43 and 44 are linear systems of algebraic equations that require the matrix A = [A_aj_^ib^] to be nonsingular in order to have a unique solution. We remark that this matrix becomes singular whenever an eigenvalue of the occupied density block is equal to an eigenvalue of the virtual density block. Although this would prevent straightforward integration of the orbital equations of motion, we have not encountered the singularity in actual simulations thus far.

The abovementioned derivation does not require unitary orbital rotations and is, therefore, applicable to TDNOCC theory. Specialization to TDOCC theory is most conveniently done by starting from the inherently real action functional^20,37^
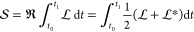
46which is required to be stationary with respect to variations of all the parameters. The expression for the Lagrangian  is identical to eq 38 with κ̂ anti-Hermitian. The Euler–Lagrange equations then take the form
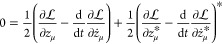
47for *z*_μ_ ∈ { κ_a_^i^, λ_μ_, τ^μ^}. The derivatives of  with respect to the complex-conjugated parameters vanish for the amplitudes λ_μ_ and τ^μ^ and, therefore, the resulting equations of motion for the amplitudes are identical to eqs 41 and 42.

Taking the derivative of  with respect to κ_i_^a^ and using eqs 43–45, we obtain the equations of motion for the orbital-rotation parameters
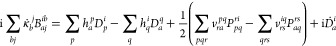
48where we have defined the hermitized one- and two-body density matrices
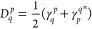
49
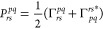
50and the matrix
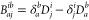
51Here, too, we face a potential singularity that we have never encountered in practical simulations thus far.

### 2.4 TDOMP2 Theory

In the spirit of the TDCC2 approximation to TDCCSD theory, we may introduce second-order approximations to TDNOCCD and TDOCCD theories, which we will designate TDNOMP2 and TDOMP2 theories, respectively, in accordance with the naming convention used in time-independent theory.^43^ The TDOMP2^26,27^ method has previously been formulated as a second-order approximation to the TDOCCD method^20,37^ by Pathak et al.^26,27^ The definition of perturbation order is analogous to that of the TDCC2 approximation to the TDCCSD method,^45^ as outlined above. Thus, the Hamiltonian is split into a zeroth-order term, *Ĥ*^(0)^(*t*) = *f̂* + *V̂*(*t*), and a first-order term, the fluctuation potential *Û* = *Ĥ*(*t*) – *f̂* – *V̂*(*t*) such that the HF reference determinant is the ground state of the zeroth-order Hamiltonian for *V̂*(*t*) → 0. The doubles amplitudes enter at the first-order level, whereas the orbital-rotation parameters are considered zeroth order in analogy with the singles amplitudes of TDCC2 theory.^45^

We start by considering non-unitary orbital rotations and introduce the κ̂-transformed operators
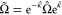
52

The TDNOMP2 Lagrangian is defined by truncating the cluster operators at the doubles level and retaining terms up to quadratic in (λ, τ, *u*) in the TDNOCC Lagrangian 38
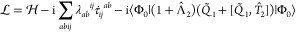
53

The TDNOMP2 Hamilton function  becomes
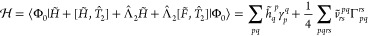
54where *h̃*_*q*_^p^, *ṽ*_*rs*_^pq^ are matrix elements transformed according to eqs 34 and 35. The operator *F̃* is given by
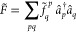
55where
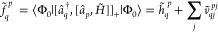
56

The non-zero matrix elements of the TDNOMP2 one- and two-body density matrices γ, Γ are given by
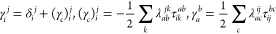
57
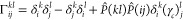
58
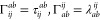
59
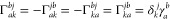
60

The equations of motion now follow from the Euler–Lagrange equations, with the Lagrangian given by eq 53. Taking the required derivatives and the κ̂ → 0 limit we find the equations of motion for the amplitudes
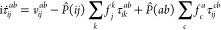
61
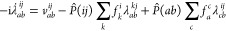
62

The time dependence of the orbital-rotation parameters in the κ̂ → 0 limit takes the same form as eqs 43 and 44, with density matrices given by eqs 57–60. The explicit insertion of non-zero matrix elements yields
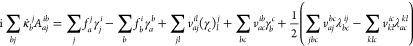
63
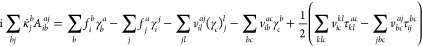
64

We can now obtain the TDOMP2 equations from the TDNOMP2 equations. The action functional takes the form of eq 46, which  is equivalent to the expression given by eq 53 with κ̂ = −κ̂^†^ and the equations of motion are obtained from the Euler-Lagrange eq 47. Because the derivatives of the Lagrangian with respect to the complex-conjugated amplitudes are zero, the equations of motion for the amplitudes are equivalent to eqs 61 and 62. However, because the orbital transformation is orthonormal, *h*, *u*, and *f* are Hermitian, and it follows that the equation for λ_*ab*_^ij^ is just the complex conjugate of that for τ_*ij*_^ab^ such that
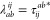
65and, thus, it is sufficient to solve only one of the two sets of amplitude equations. This simplification arises from the unitarity of the orbital rotations and is not obtained within neither TDCC2 nor TDNOMP2 theory. In addition, it follows that the one- and two-body density matrices given by eqs 57–60 are Hermitian, that is,
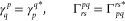
66

From the Euler-Lagrange equation, we then find that the equation of motion for κ_a_^i^ is given by
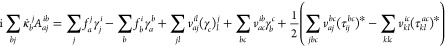
67

Note that in contrast to the TDOCC equations, there is no need to explicitly hermitize the density matrices, as they are already Hermitian within TDOMP2 theory.

### 2.5 Optical Properties from RT Simulations

In order to extract linear and nonlinear optical properties from RT time-dependent simulations, we subject an atom or molecule, initially in its (electronic) ground state, to a time-dependent electric field . The semiclassical interaction operator in the electric-dipole approximation in the length gauge is given by
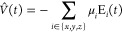
68where μ_*i*_ is the *i*th Cartesian component of the electric dipole moment operator. The shape, frequency, and strength of the electric field determine which properties may be extracted from time-dependent simulations.

Linear (one-photon) absorption spectra can be computed by using a weak electric field impulse to induce transitions from the electronic ground state to all electric-dipole allowed excited states of the system,^47,48^ including core excitations and valence excitations. Such an electric field kick is represented by the delta pulse , which we discretize by means of the box function
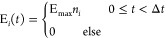
69where  is the strength of the field, *n*_*i*_ is the *i*th Cartesian component of the real unit polarization vector *n⃗*, and Δ*t* is the time step of the simulation.

The absorption spectrum is computed from the relationship
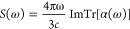
70where the frequency-dependent dipole polarizability tensor **α**(ω) is obtained from the Fourier transform of the induced dipole moment
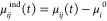
71Here, μ_*ij*_^ind^(*t*) is the *i*th component of the induced dipole moment with the field polarized in the direction *j* ∈ {*x*, *y*, *z*}, μ_*i*_^0^ is the *i*th component of the permanent dipole moment, and μ_*ij*_(*t*) is computed as the trace of the dipole matrix in the orbital basis and the effective one-body density matrix (in the same basis). In practice, we only compute finite signals at discrete points in time, forcing us to use the fast Fourier transform (FFT) algorithm. In order to avoid artifacts arising from the periodicity of the FFT algorithm, we premultiply the dipole signal with the exponential damping factor exp(−γ*t*)
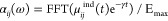
72where γ > 0 is chosen such that the induced dipole moment vanishes at the end of the simulation. This choice of the damping factor artificially broadens the excited energy levels, producing Lorentzian line shapes in the computed spectra.

Also, dynamic polarizabilities and hyperpolarizabilities can be extracted from RT time-dependent simulations using the method described by Ding et al.^49^ Suppose that the system under consideration interacts with a weak, adiabatically switched-on monochromatic electric field
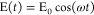
73where ω is the frequency and  is the amplitude of the field. The dipole moment can then be written as a series expansionin the electric field strength,
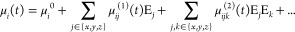
74provided that  is sufficiently small and ω belongs to a transparent spectral region of the system at hand. The time-dependent dipole response functions μ_*ij*_^(1)^(*t*) and μ_*ijk*_^(2)^(*t*) can be expressed as
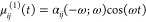
75
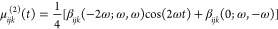
76where α_*ij*_, β_*ijk*_ are Cartesian components of the polarizability and first hyperpolarizability tensors, respectively. The “diagonal” elements μ_*ij*_^(1)^, μ_*ijj*_^(2)^ of the dipole response functions can be calculated from the time-dependent signal using the four-point central difference formulas
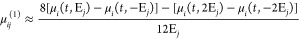
77
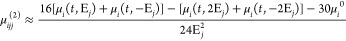
78with  being the *i*th component of the time-dependent dipole moment when a cosine field with a strength of  in the ±*j*th direction is applied. Finally, the polarizabilities and first hyperpolarizabilities are determined by performing a curve fit of the dipole response functions computed with finite differences to the analytical forms given by eqs 75 and 76.

In practice, it is infeasible to adiabatically switch on the electric field. This is circumvented by Ding et al.^49^ by turning on the field with a linear ramping envelope lasting for one optical cycle
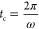
79

The electric field is then given by
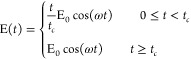
80and the curve fit is performed only on the part of the signal computed after the ramp. Furthermore, Ding et al. suggest a total simulation time of three to four optical cycles after the ramp and that field strengths in the range  are used.

## 3 Results and Discussion

In order to assess optical properties extracted from the RT TDOMP2 method, we compute absorption spectra, polarizabilities, and first hyperpolarizabilities for the 10-electron systems Ne, HF, H_2_O, NH_3_, and CH_4_. To the best of our knowledge, response theory has neither been derived nor implemented for the OMP2 method and, therefore, we compare results from TDOMP2 simulations with those extracted from RT TDCCSD and TDCC2 simulations and with results from CCSD and CC2 response theory (LRCCSD/LRCC2).^35,45^ We only compute polarizabilities and hyperpolarizabilities using the TDCC2-b method because Kats et al.^46^ found that the effect of the fully *T*_1_-transformed Fock operator on excitation energies is negligible.

For Ne, we use the d-aug-cc-pVDZ basis set in order to compare with Larsen et al.,^50^ while for the remaining molecules, we use the aug-cc-pVDZ basis set.^51−53^ Basis set specifications were downloaded from the Basis Set Exchange,^54^ and the molecular geometries used are given in the Supporting Information.

The RT simulations and correlated ground-state optimizations are carried out with a locally developed code described in previous publications^21,29,30^ using Hamiltonian matrix elements and HF orbitals computed with the PySCF package.^55^ The CCSD and CC2 ground states are computed with the direct inversion in the iterative subspace (DIIS)^56^ procedure, and the OMP2 ground state with the algorithm described by Bozkaya et al.^43^ with the diagonal approximation of the Hessian. The convergence threshold for the residual norms is set to 10^–10^. The ground-state energies and non-zero permanent dipole moments for the systems considered are given in the Supporting Information. The CCSD and CC2 linear and quadratic response calculations are performed with the Dalton quantum chemistry package.^57,58^

The TDOMP2, TDCCSD, TDCC2, and TDCC2-b equations of motion are integrated using the symplectic Gauss-Legendre integrator.^21,59^ For all cases, the integration is performed with a time step Δ*t* = 0.01 a.u. using the sixth-order (*s* = 3) Gauss–Legendre integrator and a convergence threshold of 10^–10^ (residual norm) for the fixed-point iterations. In all the RT simulations, the ground state is taken as the initial state of the system, and we use a closed-shell spin-restricted implementation of the equations. Also, the response calculations are performed in the closed-shell spin-restricted formulation.

### 3.1 Absorption Spectra

Absorption spectra are computed as described in Section 2.5 with the electric-field impulse of eq 69. The field strength is , which is small enough to ensure that only transitions from the ground state to dipole-allowed excited states occur, while strong enough to induce numerically significant oscillations. The induced dipole moment is recorded at each of 100 000 time steps after the application of the impulse, yielding a spectral resolution of about 0.006 a.u. (0.163 eV) in the FFT of eq 72. The damping parameter is γ = 0.00921 a.u. (0.251 eV), which implies that the full width at half maximum of the Lorentzian absorption lines is roughly 50% greater than the spectral resolution. Hence, very close-lying resonances will appear as a single broader absorption line, possibly with “shoulders”.

The quality of TDOMP2 absorption spectra can be assessed by comparison with the well-known and highly similar TDCC2 theory (see Supporting Information for a validation of the TDCC2 spectra by comparison with LRCC2 spectra in the range from 0 to 930 eV), the essential difference between the two methods being how orbital relaxation is treated. In general, the LRCC2 theory provides excellent valence excitation energies, often better than those of LRCCSD theory, for states with predominant single-excitation character; see, for example, the benchmark study by Schreiber et al.^60^ Preliminary and rather limited tests of excitation energies computed with NOCC theory revealed virtually no effect of the different orbital relaxation treatments^38^ and, therefore, one might expect only minor deviations between TDOMP2 and TDCC2 absorption spectra, at least in the valence regions. For a full comparison of the two methods, we will not limit ourselves to selected valence-excited states but rather compare the complete spectra up to core excitations, which are also activated by the broad-band electric-field impulse. This implies that we also compare unphysical spectral lines above the ionization threshold, which arise artificially from the use of an incomplete basis set that ignores the electronic continuum. Furthermore, we do not use proper core-correlated basis sets for describing core excitations, nor do we make any attempt at properly separating the core excitations from high-lying artificial valence excitations. Hence, no direct comparison with experimental data will be performed in this work. We instead refer to refs (24) and (61), where experimental near-edge X-ray absorption spectra are compared with those computed with a range of LRCC and EOM-CC methods and large basis sets for systems studied in this work. Importantly, the direct comparison of TDCC2 and TDOMP2 absorption spectra will indicate the effects of fully bivariational, time-dependent orbitals on core excitations where orbital relaxation is expected to play a key role—see, for example, the discussion by Park et al.^24^ for systems also considered in the present work.

**Figure 1:**
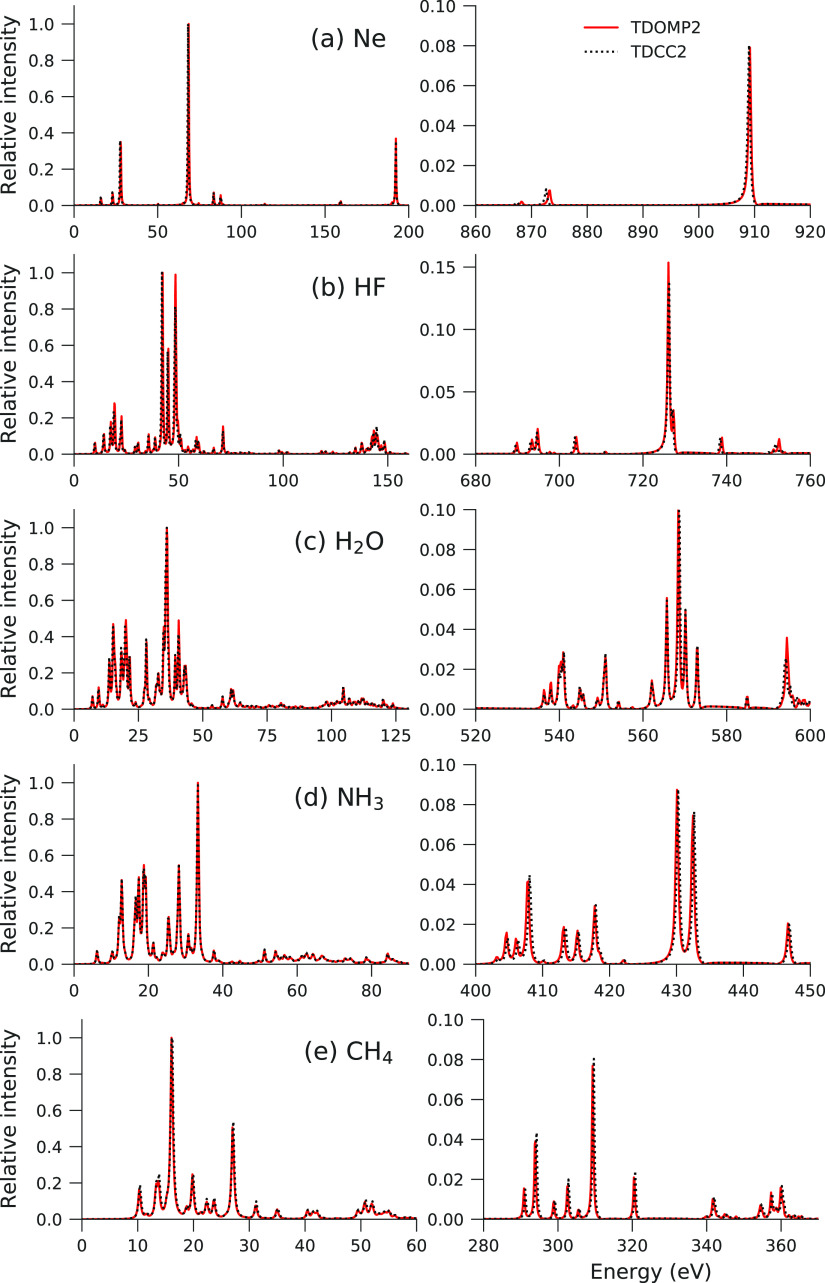
Absorption spectra computed with TDOMP2 and TDCC2 for Ne, HF, H_2_O, NH_3_, and CH_4_.

In Figure 1 we have plotted the TDOMP2 and TDCC2 electronic absorption spectra up to and including the core region.

Although deviations between the TDOMP2 and TDCC2 spectra are visible, the two methods yield very similar results both in the valence region and in the core region. The excitation energies identified from the simulated spectra by automated peak detection are reported in Table 1 for the dipole-allowed states below 30 eV and confirm the close agreement between TDOMP2 and TDCC2 theories.

**Table 1:** Dipole-Allowed Excitation Energies (in eV) below 30 eV Extracted from TDOMP2 and TDCC2 Simulations

	TDOMP2	TDCC2		TDOMP2	TDCC2		TDOMP2	TDCC2
H_2_O	7.17	7.17	NH_3_	6.15	6.15	CH_4_	10.25	10.42
	9.56	9.56		7.52	7.69		11.61	11.61
	11.10	11.10		10.25	10.25		13.32	13.49
	13.66	13.66		12.13	12.13		13.66	13.83
	15.20	15.20		12.81	12.81		16.06	16.23
	18.45	18.28		16.57	16.57		18.79	18.79
	20.15	19.81		17.42	17.42		19.81	19.81
	21.69	21.52		18.79	18.79		21.35	21.35
	23.91	23.74		19.30	19.13		22.38	22.38
	27.33	27.33		21.35	21.35		23.57	23.74
	28.18	28.01		22.20	22.20		26.99	27.16
Ne	16.06	15.88		23.91	23.91	HF	10.08	9.91
	23.06	22.89		25.45	25.28		14.35	14.18
	27.84	27.50		26.82	26.82		19.30	18.96
				28.18	28.18		22.72	22.54
				29.21	29.21		24.25	24.08
							29.21	29.04

The greatest deviations are found for the HF molecule, especially for the intensities. Some intensity deviations are expected, as the TDOMP2 method is gauge invariant (in the complete basis set limit), while the TDCC2 theory is not,^37,38^ which is bound to influence transition moments but not necessarily excitation energies. In the core regions, we note that the spectra of H_2_O, NH_3_, and CH_4_ agree qualitatively with the core spectra obtained by Park et al.^24^ from the TD-EOM-CCSD theory. Keeping in mind that the large deviations between LRCC2/LRCCSD and experimental core excitation energies are ascribed to missing orbital-relaxation effects, it is intriguing to observe that the fully bivariational orbital evolution included in the TDOMP2 theory hardly affects the core spectra relative to TDCC2 theory. Using automated peak detection, we find that the differences in excitation energies in the core region between the TDOMP2 and TDCC2 spectra are within 1–2 times the spectral resolution. Because the error of LRCC2 core excitation energies is typically several eV, we conclude that the orbital relaxation provided by TDOMP2 theory is not sufficient to significantly improve the agreement with experimental results. This observation calls for further investigations with larger basis sets, higher resolution (longer simulation times), and full inclusion of double excitations (the TDOCCD and TDNOCCD methods).

### 3.2 Polarizabilities and First Hyperpolarizabilities

Polarizabilities and first hyperpolarizabilities are computed using an electric field given by eq 80. After the initial one-cycle ramp, we propagate for three optical cycles. The first- and second-order time-dependent dipole response functions are computed by finite differences according to eqs 77 and 78, with the first optical cycle of the time evolution discarded because of the ramping. We then perform least-squares fitting^62^ of the time-domain dipole response functions to the form of eqs 75 and 76, obtaining frequency-dependent polarizabilities and hyperpolarizabilities. For all the systems, we use the field strengths  to compute the dipole derivatives using finite differences.

The diagonal elements of the frequency-dependent polarizability tensor extracted from TDCCSD, TDOMP2, TDCC2, and TDCC2-b simulations for Ne, HF, H_2_O, NH_3_, and CH_4_ are listed in Table 2 along with results from LRCCSD and LRCC2 theories.

**Table 2:** Polarizabilities (a.u.) of Ne, HF, H_2_O, NH_3_, and CH_4_ Extracted from TDCCSD, TDOMP2, TDCC2, and TDCC2-b Simulations^a^

Ne	ω (a.u.)	0.1	0.2	0.3	0.4	0.5
	LRCCSD	2.74	2.83	3.01	3.38	4.23
	TDCCSD	2.74	2.83	3.03	3.49	4.76
	TDOMP2	2.77	2.87	3.07	3.58	4.99
	LRCC2	2.86	2.96	3.18	3.59	4.74
	TDCC2	2.87	2.98	3.19	3.75	5.29
	TDCC2-b	2.86	2.97	3.18	3.73	5.26

		0.1		0.2		0.3	
HF	ω (a.u.)	α_*yy*_	α_*zz*_	α_*yy*_	α_*zz*_	α_*yy*_	α_*zz*_
	LRCCSD	4.44	6.41	4.83	6.83	6.19	7.73
	TDCCSD	4.45	6.41	4.84	6.83	6.72	7.84
	TDOMP2	4.56	6.49	5.03	6.94	7.71	7.96
	LRCC2	4.70	6.78	5.20	7.25	7.24	8.29
	TDCC2	4.75	6.85	5.28	7.36	8.54	8.45
	TDCC2-b	4.72	6.79	5.24	7.28	8.42	8.36

		0.0428			0.0656			0.1		
H_2_O	ω (a.u.)	α_*xx*_	α_*yy*_	α_*zz*_	α_*xx*_	α_*yy*_	α_*zz*_	α_*xx*_	α_*yy*_	α_*zz*_
	LRCCSD	8.78	9.93	9.11	8.89	9.99	9.19	9.18	10.14	9.37
	TDCCSD	8.78	9.93	9.11	8.90	10.00	9.19	9.19	10.14	9.37
	TDOMP2	9.16	10.06	9.34	9.29	10.13	9.42	9.62	10.27	9.63
	LRCC2	9.41	10.43	9.63	9.55	10.50	9.71	9.91	10.66	9.92
	TDCC2	9.51	10.56	9.74	9.65	10.63	9.83	10.01	10.79	10.06
	TDCC2-b	9.44	10.47	9.66	9.58	10.54	9.74	9.94	10.71	9.97

		0.0428		0.0656		0.1	
NH_3_	ω (a.u.)	α_*yy*_	α_*zz*_	α_*yy*_	α_*zz*_	α_*yy*_	α_*zz*_
	LRCCSD	13.10	15.04	13.20	15.35	13.44	16.15
	TDCCSD	13.10	15.05	13.20	15.36	13.45	16.15
	TDOMP2	13.23	15.60	13.34	15.98	13.59	16.95
	LRCC2	13.56	15.86	13.67	16.21	13.92	17.15
	TDCC2	13.72	16.03	13.83	16.43	14.10	17.40
	TDCC2-b	13.64	15.93	13.75	16.32	14.01	17.28

CH_4_	ω (a.u.)	0.0656	0.1	0.2
	LRCCSD	17.05	17.39	19.55
	TDCCSD	17.05	17.39	19.58
	TDOMP2	17.18	17.53	19.79
	LRCC2	17.49	17.84	20.08
	TDCC2	17.69	18.05	20.34
	TDCC2-b	17.61	17.96	20.25

a The LRCCSD and LRCC2 results for Ne and HF are from ref (50)., and the remaining LRCCSD and LRCC2 results are computed with the Dalton quantum chemistry program (ref (57).).

All the three diagonal elements are identical by symmetry for Ne and CH_4_, α_*xx*_ = α_*yy*_ for HF and NH_3_, and off-diagonal elements vanish for all the systems considered here. The polarizability diverges at the (dipole-allowed) excitation energies and, therefore, we select frequencies below the first dipole-allowed transition in Table 1 (roughly 0.6 a.u. for Ne, 0.4 a.u. for HF, 0.3 a.u. for H_2_O, 0.2 a.u. for NH_3_, and 0.4 a.u. for CH_4_).

The benchmark study by Larsen et al.^50^ indicated that the LRCCSD theory yields accurate static and dynamic polarizabilities, although triple excitations are needed to obtain results very close to the FCI theory, whereas results from the LRCC2 theory are significantly less accurate. Our results in Table 2 confirm this finding in the sense that TDCC2 (and LRCC2) results are quite far from the corresponding TDCCSD (and LRCCSD) results. We also note that TDCCSD and LRCCSD results agree to a much greater extent than the results from TDCC2 and LRCC2 theories.

Unfortunately, we have not been able to identify the source of this behavior in the TDCC2 model. The agreement between the results from simulations and from response theory generally worsens as the frequency approaches the lowest-lying dipole-allowed transition. In this “semitransparent” regime, the assumptions of linear response theory are violated and the first-order time-dependent induced dipole moment cannot be described as the simple function in eq 75.

**Figure 2:**
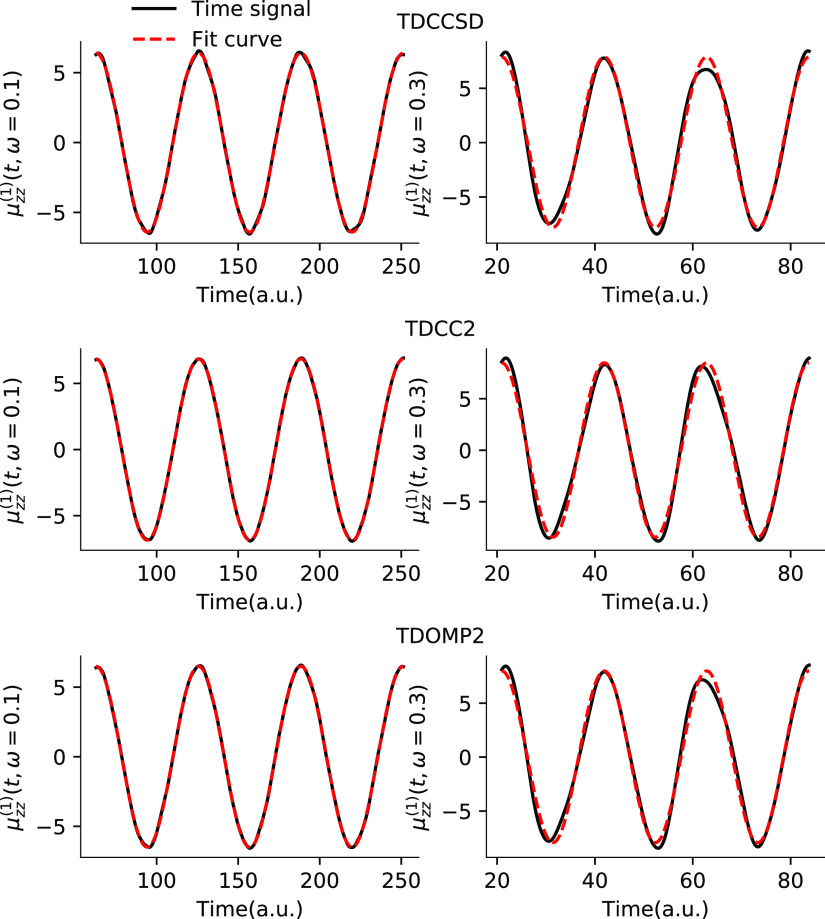
*zz*-component of the first-order dipole responses for HF at ω = 0.1 a.u. and ω = 0.3 a.u. from TDCCSD, TDCC2, and TDOMP2 simulations.

This is confirmed by the plots of simulated time signals and the least-squares fits in Figure 2 where the former clearly can only be accurately described by eq 75 at sufficiently low (transparent) frequencies. The TDCC2 least-squares fits, however, do not appear worse than those of TDCCSD or TDOMP2 theory. Hence, larger deviations from the form in eq 75 cannot explain the discrepancies between TDCC2 and LRCC2 results.

**Figure 3:**
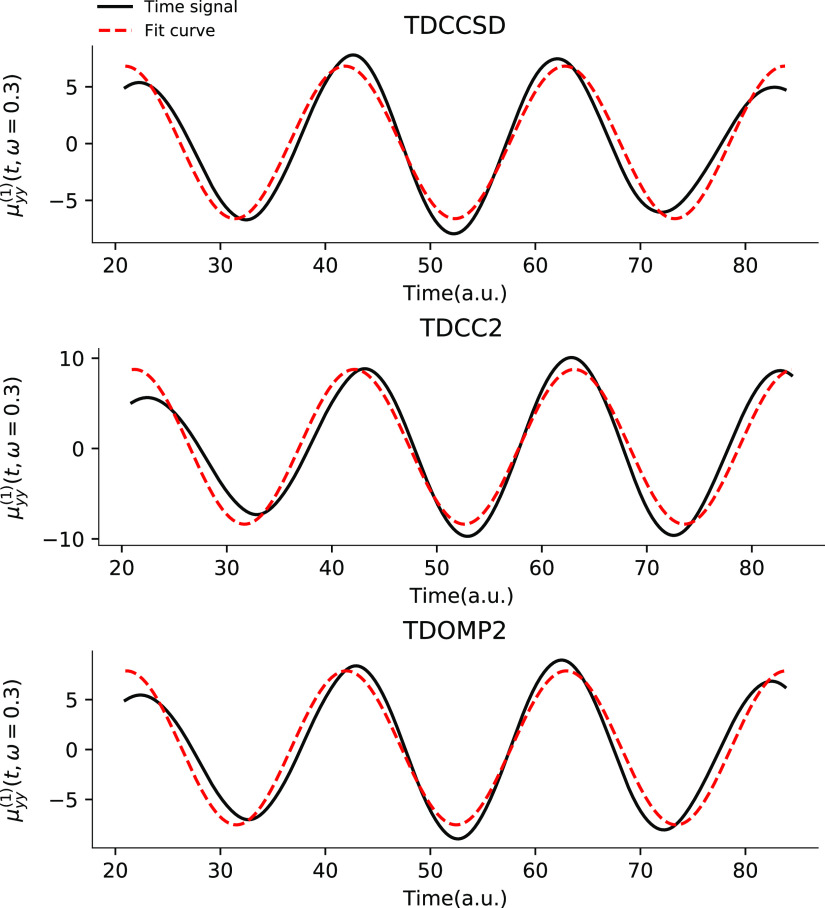
*yy*-component of the first-order dipole responses for HF at ω = 0.3 a.u. from TDCCSD, TDCC2, and TDOMP2 simulations.

Furthermore, we note the relatively large discrepancy between the LRCCSD and TDCCSD results for the HF molecule at ω = 0.3 a.u. and the Ne atom at ω = 0.4 a.u. and ω = 0.5 a.u. In these cases, the first-order response function extracted from the time-dependent simulations (77) for all the methods considered does not agree with the assumption of a pure cosine wave (75), as shown in Figure 3 for the HF molecule. The source of deviation is a combined effect of proximity to a pole, nonadiabatic effects arising from ramping up the field over a single cycle, and the absence of higher-order corrections in the finite-difference expressions for the response functions.^49^ This is also likely to be the source of the irregular behavior of α_*yy*_ computed with the TDCC2 and TDCC2-b methods.

**Figure 4:**
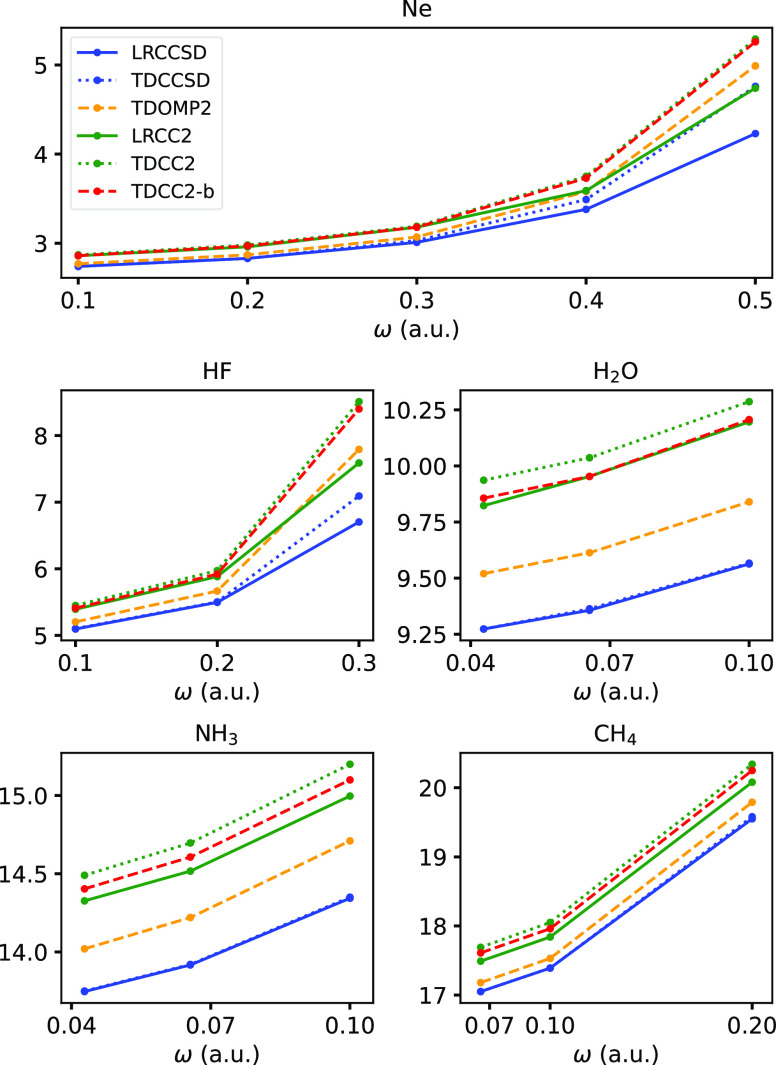
Isotropic polarizabilities extracted from TDCC2, TDCC2-b, TDOMP2, and TDCCSD simulations, and from LRCC2 and LRCCSD calculations.

Interestingly, we observe that polarizabilities from the TDOMP2 theory are generally in better agreement with the TDCCSD values than those from TDCC2 (and LRCC2) theory. This trend is particularly evident from Figure 4 where we have plotted the dispersion of the isotropic polarizability, α_iso_ = (α_*xx*_ + α_*yy*_ + α_*zz*_)/3.

Keeping in mind the similarity between the TDOMP2 and TDCC2 spectra, the pronounced difference between TDOMP2 and TDCC2 polarizabilities is somewhat surprising. It is, however, in agreement with the observation by Larsen et al.^50^ that orbital relaxation has a sizeable impact on polarizabilities within CC theory, albeit not always improving the results relative to FCI calculations. Only static polarizabilities were considered by Larsen et al.^50^ because the orbital relaxation—formulated as a variational HF constraint within conventional CC response theory—leads to spurious uncorrelated poles in the response functions, making it useless for dynamic polarizabilities. The orbitals are treated as fully bivariational variables within the TDOMP2 theory and, consequently, spurious poles are avoided.^37^ Our results, therefore, seem to indicate that a fully bivariational treatment of orbital relaxation is beneficial for polarizability predictions. The partial orbital relaxation included in the TDCC2-b method does not yield equally good polarizabilities. In most cases, the results are nearly identical to the TDCC2 ones, except for the H_2_O and NH_3_ molecules, where the TDCC2-b polarizabilities are closer to the LRCC2 results, see Figure 4.

In Table 3 we list frequency-dependent first hyperpolarizabilities for HF, H_2_O, and NH_3_. Only the nonvanishing diagonal components of the practically most important response tensors corresponding to optical rectification (OR), β_*iii*_^OR^ = β_*iii*_(0, ω, −ω), and second harmonic generation (SHG), β_*iii*_^SHG^ = β_*iii*_(−2ω, ω, ω), are computed. Formally expressable as a double summation over all the excited states, the first hyperpolarizability generally requires a high-level description of electron correlation effects for accurate calculations.^63^ This is reflected in our results by the relatively large difference between the TDCC2 and TDCCSD methods.

**Table 3:** First Hyperpolarizabilities (a.u.) of HF, H_2_O, and NH_3_ from TDCCSD, TDOMP2, TDCC2, and TDCC2-b Simulations^a^

		0.1		0.2		0.3	
HF	ω (a.u.)	β_*zzz*_^OR^	β_*zzz*_^SHG^	β_*zzz*_^OR^	β_*zzz*_^SHG^	β_*zzz*_^OR^	β_*zzz*_^SHG^
	LRCCSD	12.81	14.38	15.28	29.40	21.86	–229.70
	TDCCSD	12.89	14.45	15.63	29.32	25.11	–73.94
	TDOMP2	13.05	14.66	15.21	28.16	24.98	–65.73
	LRCC2	15.52	17.52	18.69	37.67	27.35	–51.78
	TDCC2	16.53	18.63	19.40	36.39	32.11	–61.17
	TDCC2-b	15.32	17.26	17.95	33.56	29.76	–64.95

		0.0428		0.0656		0.1	
H_2_O	ω (a.u.)	β_*zzz*_^OR^	β_*zzz*_^SHG^	β_*zzz*_^OR^	β_*zzz*_^SHG^	β_*zzz*_^OR^	β_*zzz*_^SHG^
	LRCCSD	–9.11	–9.59	–9.43	–10.72	–10.25	–14.52
	TDCCSD	–9.14	–9.62	–9.50	–10.78	–10.47	–14.69
	TDOMP2	–9.92	–10.49	–10.33	–11.80	–11.57	–17.63
	LRCC2	–12.39	–13.12	–12.87	–14.83	–14.11	–20.76
	TDCC2	–13.63	–14.42	–14.17	–16.18	–15.75	–23.70
	TDCC2-b	–11.89	–12.58	–12.38	–14.15	–13.84	–21.01

		0.0428				0.0656			
NH_3_	ω (a.u.)	β_*yyy*_^OR^	β_*yyy*_^SHG^	β_*zzz*_^OR^	β_*zzz*_^SHG^	β_*yyy*_^OR^	β_*yyy*_^SHG^	β_*zzz*_^OR^	β_*zzz*_^SHG^
	LRCCSD	–14.90	–15.50	23.90	28.02	–15.30	–16.88	26.57	40.49
	TDCCSD	–14.94	–15.59	24.20	28.45	–15.47	–17.27	27.35	41.94
	TDOMP2	–15.64	–16.42	30.66	36.26	–15.81	–17.39	35.50	58.38
	LRCC2	–16.69	–17.40	33.80	39.87	–17.16	–19.01	37.72	58.61
	TDCC2	–17.32	–18.13	35.80	41.90	–17.51	–19.17	41.24	66.61
	TDCC2-b	–17.00	–17.79	32.60	38.26	–17.19	–18.81	37.80	61.67

a Notation: β_*iii*_^OR^ = β_*iii*_(0; ω, −ω) and β_*iii*_^SHG^ = β_*iii*_(−2ω; ω, ω). The LRCCSD and LRCC2 results for HF are taken from Larsen et al.^50^

While β_*iii*_^OR^ is singular when the magnitude of the radiation frequency ω equals the excitation energy of the molecule, β_*iii*_^SHG^ has an additional set of poles at half the excitation energies. The β_*zzz*_^SHG^ results at ω = 0.3 a.u. for the HF molecule in Table 3 are past the first pole and, hence, the sign has changed compared with the SHG results at lower frequencies. The large negative value of β_*zzz*_^SHG^ at ω = 0.3 a.u. obtained with the LRCCSD method for the HF molecule is due to proximity to two dipole-allowed, *z*-polarized excitations at 0.598 a.u. (oscillator strength 0.005) and at 0.532 a.u. (oscillator strength 0.157).

**Figure 5:**
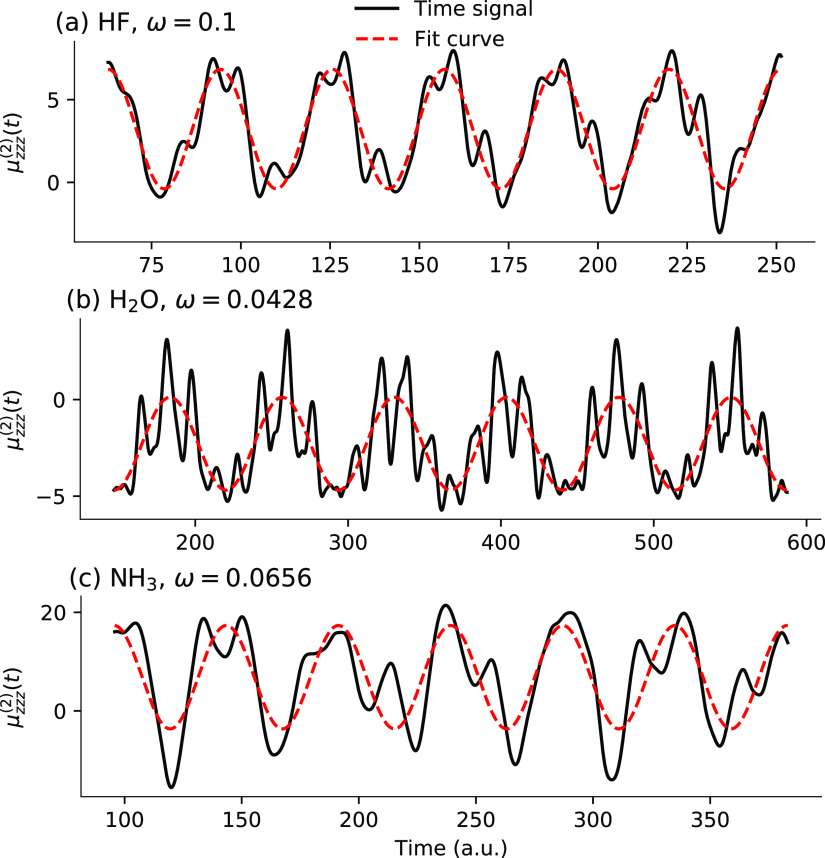
Second-order dipole responses for HF, H_2_O, and NH_3_ from TDCCSD simulations.

The agreement between RT simulations and response theory is seen to be somewhat worse than for polarizabilities, especially for frequencies closer to the pole of the hyperpolarizability. To a large extent this can be ascribed to the second-order dipole response extracted from the time-dependent simulations not being well described by the sinusoidal form of eq 76, as illustrated in Figure 5.

Analogous observations were done by Ding et al.^49^ in the context of RT-TDDFT simulations. Hence, moving on to higher-order nonlinear optical properties cannot generally be expected to provide more than a rough estimate with the present extraction algorithm. As for the polarizabilites mentioned above, we observe that the first hyperpolarizabilities obtained from TDOMP2 simulations are generally closer to TDCCSD and LRCCSD results than those from TDCC2 and LRCC2 theory. The source of the improvement over TDCC2 theory must be the bivariational orbital relaxation, although we stress that the larger differences between TDOMP2 theory and TDCCSD theory, which are particularly pronounced for NH_3_, clearly demonstrate the insufficient electron correlation treatment of the former for highly accurate predictions of nonlinear optical properties. The importance of orbital relaxation is corroborated by the TDCC2-b hyperpolarizabilities, which are somewhat closer to the TDOMP2 and TDCCSD results than the TDCC2 ones.

## 4 Concluding Remarks

In this work, we have presented a new unified derivation of TDOCC and TDNOCC theories, including the second-order approximations TDOMP2 and TDNOMP2, using exponential orbital-rotation operators and the bivariational Euler–Lagrange equations. Using five small 10-electron molecules as test cases, we have extracted absorption spectra and frequency-dependent polarizabilities and hyperpolarizabilities from TDOMP2 simulations with weak fields within the electric-dipole approximation and compared the results with those from conventional TDCCSD and TDCC2 simulations. Although the TDOMP2 absorption spectra are almost identical to the TDCC2 spectra, including in the spectral region of core excitations, the TDOMP2 polarizabilities and hyperpolarizabilities are significantly closer to the TDCCSD results than those from TDCC2 simulations, especially for frequencies comfortably away from resonances. Further corroborated by TDCC2-b simulations, our results strongly indicate that fully (bi-)variational orbital relaxation is important for frequency-dependent polarizabilities and hyperpolarizabilities, while nearly irrelevant for absorption spectra.

Combined with the observations by Pathak et al.,^26^ who found that TDOMP2 theory outperforms TDCC2 theory for strong-field many-electron dynamics, our results may serve as a motivation for further development of TDOMP2 theory. First of all, a reduced-scaling implementation of TDOMP2 theory, obtained, for example, by exploiting the sparsity of the correlating doubles amplitudes,^64^ can provide reasonably accurate results for larger systems and basis sets that are out of reach for today’s TDCC implementations. Second, an efficient implementation of OMP2 linear and quadratic response functions is warranted.

## Supporting Information Available

(Supporting information ZIP)
Algebraic expressions for the closed-shell spin-restricted OMP2 method, molecular geometries, electronic ground-state energies and electric-dipole moments, and comparison of TDCC2 absorption spectra with those from the LRCC2 theory in the range 0–930 eV (PDF)
