# Correction to “Linear and Nonlinear Optical Properties from TDOMP2 Theory”

**DOI:** 10.1021/acs.jctc.2c00830

**Published:** 2022-08-25

**Authors:** Håkon Emil Kristiansen, Benedicte Sverdrup Ofstad, Eirill Hauge, Einar Aurbakken, Øyvind Sigmundson Schøyen, Simen Kvaal, Thomas Bondo Pedersen

An error has been discovered in the equations of motion (EOMs) for the  amplitudes of the TDCC2 method, eq 22. The contributions arising from the term
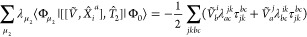
1of the TDCC2 Hamilton function, eq 21, were erronously left out. Including these contributions, eq 22 becomes
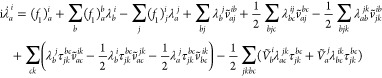
2

The missing contributions were also left out in the implementation of the TDCC2 and TDCC2-b methods. We have added the missing contributions to the implementation, which has been verified by comparing expectation values of the electric-dipole operator at nonzero electric-field strengths with those obtained by numerical differentiation (second-order central difference) of total energies. Note that energy derivatives at *nonzero* field strengths are required to detect the error.

The peak positions in the absorption spectra (i.e., the excitation energies) are unaffected by the error since the EOMs for the τ amplitudes are unchanged. The relative intensities deviate from the original results by at most 0.0015, typically much less, for all molecules studied. Errors of this magnitude are not visible in plotted spectra. The maximum error occurs for the water molecule as shown in Figure 1. Hence, the reported TDCC2 and TDCC2-b spectra are practically unaffected.

**Figure 1:**
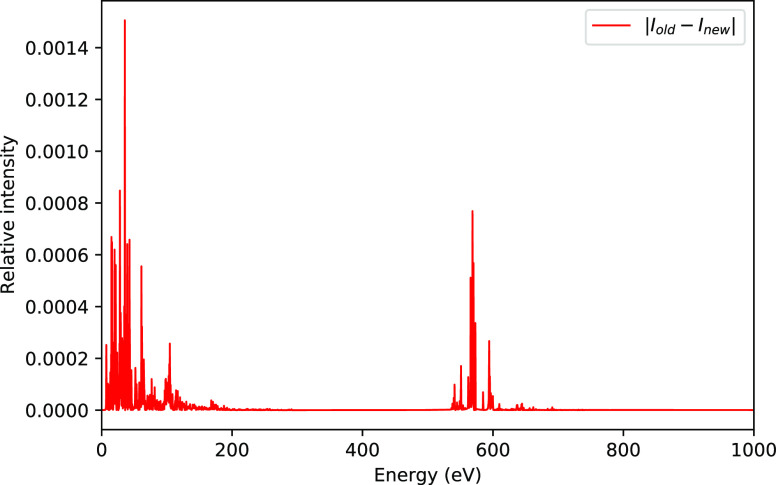
Absolute difference of the TDCC2 absorption spectra of the water molecule before (“old”) and after (“new”) correcting the EOMs for the  amplitudes.

On the other hand, the computed values of the polarizabilities and first hyperpolarizabilities change. In Table 1 and Table 2 we report updated values for the polarizabilities and first hyperpolarizabilities, respectively. We note that the TDCC2 results are now in much better agreement with the LRCC2 results than originally reported. This is also evident in Figure 4 (replacing Figure 4 in the original paper) where we have plotted the dispersion of the isotropic polarizability using the new polarizabilties from TDCC2 and TDCC2-b simulations. Also, in Figure 2 and Figure 3 we have plotted the first-order dipole response functions for the HF molecule from corrected TDCC2 simulations, which corrects the middle panels of Figure 2 and Figure 3 in the original paper, respectively.

**Table 1:** Polarizabilities (au) of Ne, HF, H_2_O, NH_3_, and CH_4_ Extracted from LRCC2, TDCC2, and TDCC2-b Simulations^a^

		ω = 0.1 au	ω = 0.2 au	ω = 0.3 au	ω = 0.4 au	ω = 0.5 au
Ne	LRCC2*	2.86	2.96	3.18	3.59	4.74
	TDCC2	2.86	2.96	3.18	3.73	5.27
	TDCC2*	2.87	2.98	3.19	3.75	5.29
	TDCC2-b	2.84	2.95	3.16	3.71	5.23
	TDCC2-b*	2.86	2.97	3.18	3.73	5.26

		ω = 0.1 au		ω = 0.2 au		ω = 0.3 au	
		α_*yy*_	α_*zz*_	α_*yy*_	α_*zz*_	α_*yy*_	α_*zz*_
HF	LRCC2*	4.70	6.78	5.20	7.25	7.24	8.29
	TDCC2	4.71	6.78	5.24	7.28	8.47	8.36
	TDCC2*	4.75	6.85	5.28	7.36	8.54	8.45
	TDCC2-b	4.68	6.72	5.20	7.20	8.35	8.27
	TDCC2-b*	4.72	6.79	5.24	7.28	8.42	8.36

		ω = 0.0428 au			ω = 0.0656 au			ω = 0.1 au		
		α_*xx*_	α_*yy*_	α_*zz*_	α_*xx*_	α_*yy*_	α_*zz*_	α_*xx*_	α_*yy*_	α_*zz*_
H_2_O	LRCC2*	9.41	10.43	9.63	9.55	10.50	9.71	9.91	10.66	9.92
	TDCC2	9.41	10.43	9.63	9.55	10.50	9.71	9.91	10.67	9.94
	TDCC2*	9.51	10.56	9.74	9.65	10.63	9.83	10.01	10.79	10.06
	TDCC2-b	9.35	10.35	9.55	9.49	10.42	9.63	9.84	10.58	9.86
	TDCC2-b*	9.44	10.47	9.66	9.58	10.54	9.74	9.94	10.71	9.97

		ω = 0.0428 au		ω = 0.0656 au		ω = 0.1 au	
		α_*yy*_	α_*zz*_	α_*yy*_	α_*zz*_	α_*yy*_	α_*zz*_
NH_3_	LRCC2*	13.56	15.86	13.67	16.21	13.92	17.15
	TDCC2	13.56	15.86	13.67	16.25	13.93	17.21
	TDCC2*	13.72	16.03	13.83	16.43	14.10	17.40
	TDCC2-b	13.48	15.76	13.59	16.15	13.84	17.09
	TDCC2-b*	13.64	15.93	13.75	16.32	14.01	17.28

		ω = 0.0656 au	ω = 0.1 au	ω = 0.2 au
CH_4_	LRCC2*	17.49	17.84	20.08
	TDCC2	17.50	17.85	20.12
	TDCC2*	17.69	18.05	20.34
	TDCC2-b	17.42	17.77	20.02
	TDCC2-b*	17.61	17.96	20.25

a The LRCC2 results for Ne and HF are from ref (2), and the LRCC2 results are computed with the Dalton quantum chemistry program (ref (3)). The calculations marked with * refers to the values reported in the original paper.

**Table 2:** First Hyperpolarizabilities (au) of HF, H_2_O, and NH_3_ from TDCCSD, TDOMP2, TDCC2, and TDCC2-b Simulations^a^

		ω = 0.1 au		ω = 0.2 au		ω = 0.3 au	
		β_*zzz*_^OR^	β_*zzz*_^SHG^	β_*zzz*_^OR^	β_*zzz*_^SHG^	β_*zzz*_^OR^	β_*zzz*_^SHG^
HF	LRCC2*	15.52	17.52	18.69	37.67	27.35	–51.78
	TDCC2	15.54	17.53	18.26	34.31	30.57	–60.52
	TDCC2*	16.53	18.63	19.40	36.39	32.11	–61.17
	TDCC2-b	14.73	16.62	17.26	32.34	28.76	–64.06
	TDCC2-b*	15.32	17.26	17.95	33.56	29.76	–64.95

		ω = 0.0428 au		ω = 0.0656 au		ω = 0.1 au	
		β_*zzz*_^OR^	β_*zzz*_^SHG^	β_*zzz*_^OR^	β_*zzz*_^SHG^	β_*zzz*_^OR^	β_*zzz*_^SHG^
H_2_O	LRCC2*	–12.39	–13.12	–12.87	–14.83	–14.11	–20.76
	TDCC2	–12.42	–13.16	–12.93	–14.83	–14.45	–22.02
	TDCC2*	–13.63	–14.42	–14.17	–16.18	–15.75	–23.70
	TDCC2-b	–11.21	–11.88	–11.69	–13.41	–13.10	–20.08
	TDCC2-b*	–11.89	–12.58	–12.38	–14.15	–13.84	–21.01

		ω = 0.0428 au				ω = 0.0656 au			
		β_*yyy*_^OR^	β_*yyy*_^SHG^	β_*zzz*_^OR^	β_*zzz*_^SHG^	β_*yyy*_^OR^	β_*yyy*_^SHG^	β_*zzz*_^OR^	β_*zzz*_^SHG^
NH_3_	LRCC2*	–16.69	–17.40	33.80	39.87	–17.16	–19.01	37.72	58.61
	TDCC2	–16.78	–17.58	33.97	39.88	–16.97	–18.59	39.26	63.96
	TDCC2*	–17.32	–18.13	35.80	41.90	–17.51	–19.17	41.24	66.61
	TDCC2-b	–16.59	–17.37	31.53	37.10	–16.77	–18.37	36.62	60.11
	TDCC2-b*	–17.00	–17.79	32.60	38.26	–17.19	–18.81	37.80	61.67

a Notation:  and . The LRCCSD and LRCC2 results for HF are taken from Larsen et al.^2^

**Figure 2:**
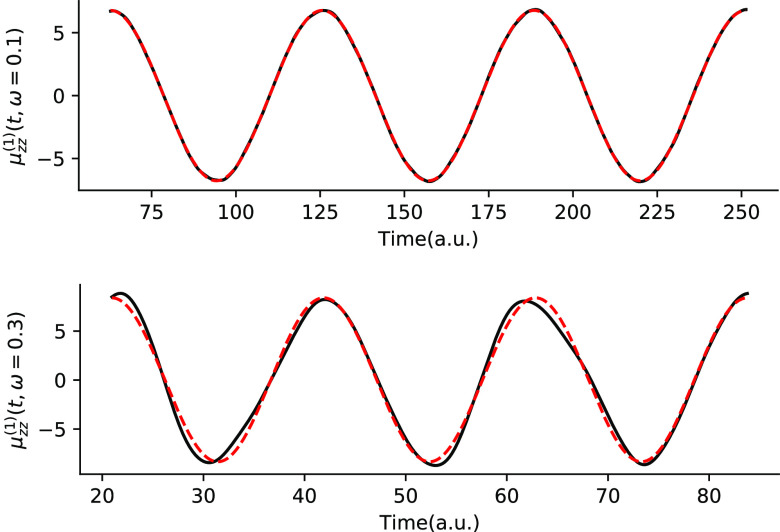
Zz-component of the first-order dipole responses for HF at ω = 0.1 au and ω = 0.3 au from corrected TDCC2 simulations.

**Figure 3:**
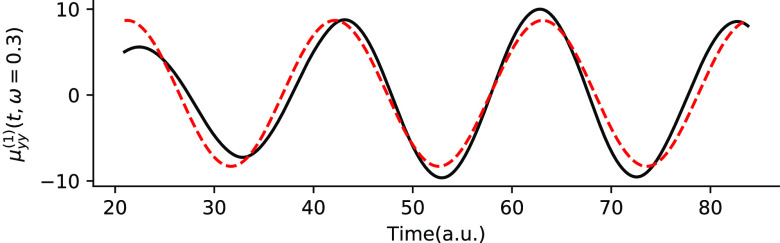
Yy-component of the first-order dipole response for HF at ω = 0.3 au from the corrected TDCC2 simulation.

**Figure 4:**
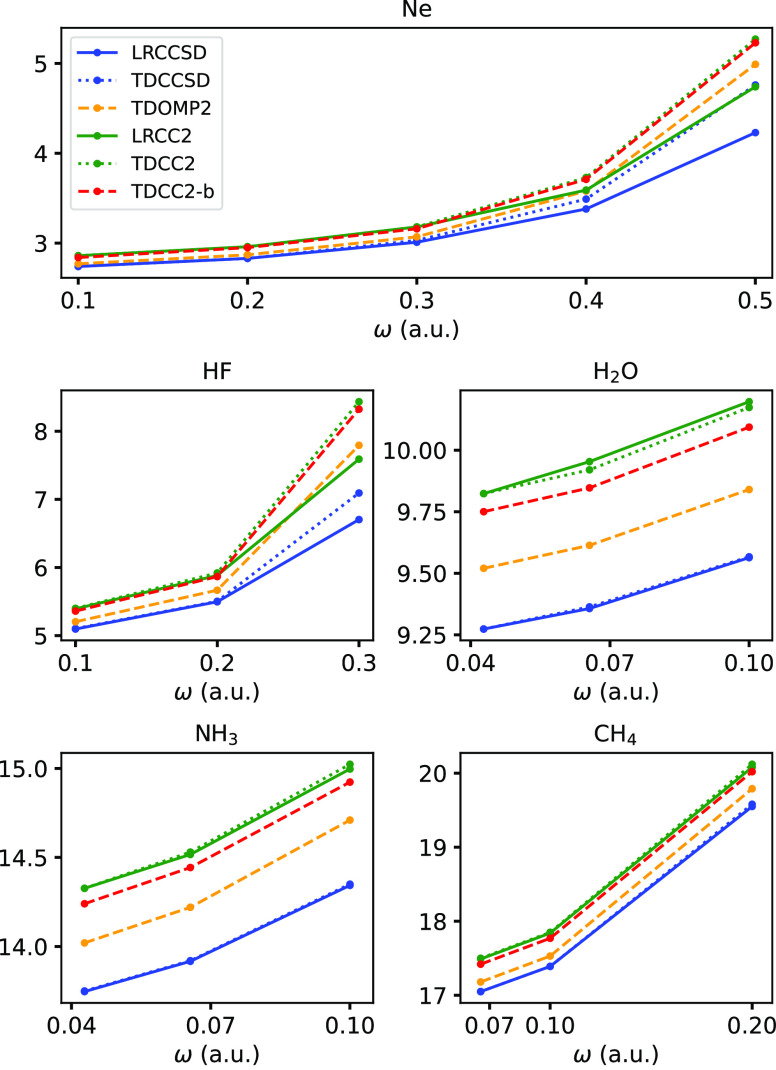
Isotropic polarizabilities extracted from TDCC2, TDOMP2, and TDCCSD simulations and from LRCC2 and LRCCSD calculations.

Finally, we emphasize that the conclusions of the original paper are unaffected by the error.
