# Supplementary figures and images for: Linear and Nonlinear Optical Properties from TDOMP2 Theory
Source: J Chem Theory Comput. 2022 Apr 18;18(6):3687–702. doi: 10.1021/acs.jctc.1c01309 (PMC9202312; doi:10.1021/acs.jctc.1c01309)

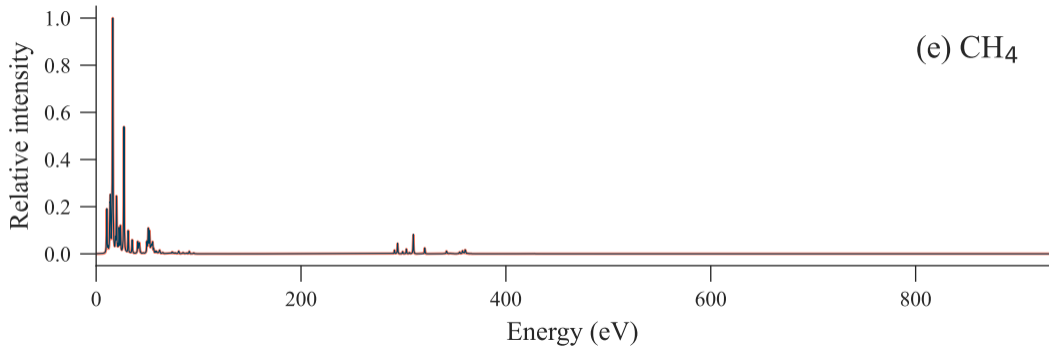

Supplement: Supplementary file 1 — ct1c01309_si_001.zip [file ct1c01309_si_001.zip › abs_spec_ch4_aug-cc-pvdz_tdcc2_vs_lrcc2.pdf]

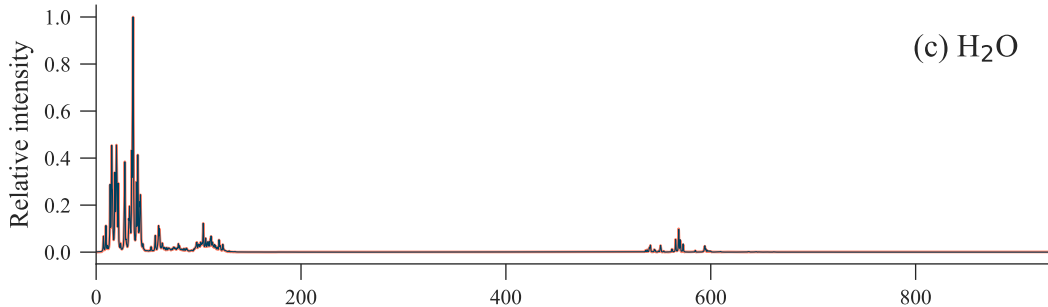

Supplement: Supplementary file 1 — ct1c01309_si_001.zip [file ct1c01309_si_001.zip › abs_spec_h2o_aug-cc-pvdz_tdcc2_vs_lrcc2.pdf]

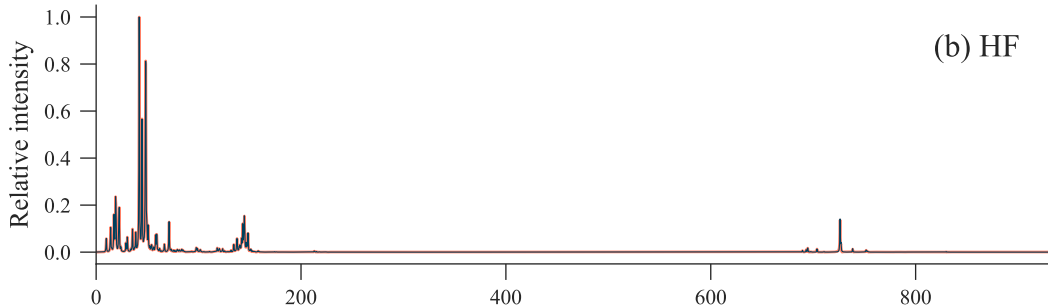

Supplement: Supplementary file 1 — ct1c01309_si_001.zip [file ct1c01309_si_001.zip › abs_spec_hf_aug-cc-pvdz_tdcc2_vs_lrcc2.pdf]

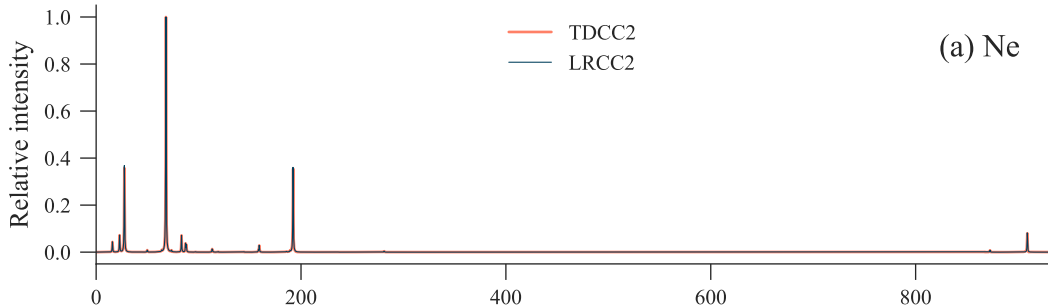

Supplement: Supplementary file 1 — ct1c01309_si_001.zip [file ct1c01309_si_001.zip › abs_spec_ne_d-aug-cc-pvdz_tdcc2_vs_lrcc2.pdf]

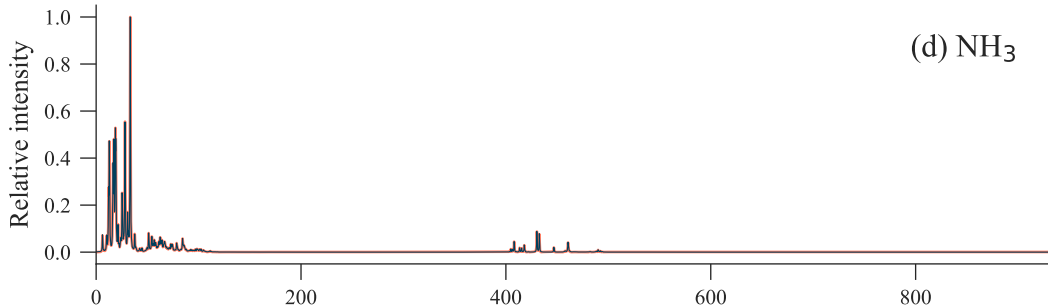

Supplement: Supplementary file 1 — ct1c01309_si_001.zip [file ct1c01309_si_001.zip › abs_spec_nh3_aug-cc-pvdz_tdcc2_vs_lrcc2.pdf]
