# Supplementary material for: Linear and Nonlinear Optical Properties from TDOMP2 Theory
Source: J Chem Theory Comput. 2022 Apr 18;18(6):3687–702. doi: 10.1021/acs.jctc.1c01309 (PMC9202312; doi:10.1021/acs.jctc.1c01309)
Supplement: Supplementary file 2 — ct1c01309_si_002.pdf [file ct1c01309_si_002.pdf]

Supporting Information for "Linear and Nonlinear Optical Properties from TDOMP2 Theory"

Håkon Emil Kristiansen,[*,†] Benedicte Sverdrup Ofstad,[†] Eirill Hauge,[†,‡] Einar Aurbakken,[†] Øyvind Sigmundson Schøyen,[¶] Simen Kvaal,[§,†] and Thomas Bondo Pedersen[*,§,†]

†*Hylleraas Centre for Quantum Molecular Sciences, Department of Chemistry, University of Oslo, N-0315 Oslo, Norway*

‡*Simula Research Laboratory, Kristian Augusts gate 23, 0164 Oslo, Norway*

¶*Department of Physics, University of Oslo, N-0316 Oslo, Norway*

§*Centre for Advanced Study at the Norwegian Academy of Science and Letters, Drammensveien 78, N-0271 Oslo, Norway*

E-mail: h.e.kristiansen@kjemi.uio.no; t.b.pedersen@kjemi.uio.no

Abstract

The Supporting Information gives algebraic expressions for the closed-shell spin-restricted OMP2 method, molecular geometries, electronic ground-state energies and dipole moments, and a comparison of TDCC2 absorption spectra with those from LRCC2 theory from 0–930 eV.

1 Closed-shell spin-restricted OMP2 expressions

We now assume closed-shell systems where each orbital is doubly occupied. Then the expression for the fock matrix is given by

$$f_q^p = h_q^p + 2u_{qj}^{pj} - u_{jq}^{pj}. \tag{1}$$

Using the following biorthogonal paramterization of $\hat{\Lambda}, \hat{T}$,

$$\hat{T}_2 = \frac{1}{2}\sum_{abij} \tau_{ij}^{ab} \hat{E}_i^a \hat{E}_j^b,$$
$$\hat{\Lambda}_2 = \frac{1}{2}\sum_{abij} \lambda_{ab}^{ij} \left(\frac{1}{3}\hat{E}_b^j \hat{E}_a^i + \frac{1}{6}\hat{E}_b^i \hat{E}_a^j\right),$$

the derivatives of the OMP2 Hamilton function w.r.t $(\lambda_{ab}^{ij}, \kappa_a^i)$ are given by

$$\frac{\partial \mathcal{H}}{\partial \lambda_{ab}^{ij}} = u_{ij}^{ab} + P_{ij}^{ab}\left(f_c^a \tau_{ij}^{cb} - f_i^k \tau_{kj}^{ab}\right), \tag{2}$$
$$\frac{\partial \mathcal{H}}{\partial \kappa_a^i} = h_i^b \gamma_b^a - h_j^a \gamma_i^j + u_{ir}^{pq} \Gamma_{pq}^{ar} - u_{rs}^{aq} \Gamma_{iq}^{rs}. \tag{3}$$

Furthermore, one can show that

$$\lambda_{ab}^{ij} = 2(2\tau_{ij}^{ab} - \tau_{ji}^{ab})^*. \tag{4}$$

The expressions for the one- and two-body density matrices are given by

$$\gamma_i^j = 2\delta_i^j + (\gamma_c)_i^j, \quad (\gamma_c)_i^j = -\lambda_{ab}^{kj} \tau_{ki}^{ab} \tag{5}$$

$$\gamma_a^b = \lambda_{ac}^{ij} \tau_{ij}^{bc} \tag{6}$$

and

$$\Gamma_{ij}^{kl} = 4\delta_i^k\delta_j^l - 2\delta_i^l\delta_j^k + \hat{P}_{ij}^{kl}\left(-2\delta_i^k(\gamma_c)_j^l + \delta_j^k(\gamma_c)_i^l\right), \tag{7}$$

$$\Gamma_{ij}^{ab} = 2(2\tau_{ij}^{ab} - \tau_{ji}^{ab}), \tag{8}$$

$$\Gamma_{ab}^{ij} = \lambda_{ab}^{ij} = 2(2\tau_{ab}^{ij} - \tau_{ab}^{ji})^* = (\Gamma_{ij}^{ab})^*, \tag{9}$$

$$\Gamma_{ia}^{jb} = 2\delta_i^j\gamma_a^b = \Gamma_{ai}^{bj}, \tag{10}$$

$$\Gamma_{ia}^{bj} = -\delta_i^j\gamma_a^b = \Gamma_{ai}^{jb}. \tag{11}$$

2 Molecular geometries

Table 1 lists the molecular geometries used in the main article.

Table 1: Molecular geometries (Cartesian coordinates, in Bohr).

HF	H	0.0	0.0	0.0
	F	0.0	0.0	1.732 879 5
H_2O	O	0.0	0.0	−0.123 909 356 3
	H	0.0	1.429 937 284 0	0.983 265 756 7
	H	0.0	−1.429 937 284 0	0.983 265 756 7
NH_3	N	0.0	0.0	0.2010
	H	0.0	1.7641	−0.4690
	H	1.5277	−0.8820	−0.4690
	H	−1.5277	−0.8820	−0.4690
CH_4	C	0.0	0.0	0.0
	H	1.2005	1.2005	1.2005
	H	−1.2005	−1.2005	1.2005
	H	−1.2005	1.2005	−1.2005
	H	1.2005	−1.2005	−1.2005

3 Ground-state energies and electric dipole moments

Table 2 lists ground-state energies and the z-component of the electric dipole moments computed with the d-aug-cc-pVDZ basis set for Ne and the aug-cc-pVDZ basis set for the remaining molecules.

Table 2: Ground-state energies (a.u.) and electric dipole moments (a.u.) for Ne, HF, H_2O, NH_3, CH_4.

		E_0	μ_z
Ne	CCSD	−128.708 821 187 1	0.0
	OMP2	−128.707 014 780 2	0.0
	CC2	−128.707 468 246 7	0.0
HF	CCSD	−100.261 508 470 8	−0.703 237 143 6
	OMP2	−100.260 190 513 7	−0.700 524 169 9
	CC2	−100.260 558 712 4	−0.689 047 761 5
H_2O	CCSD	−76.270 767 643 3	0.729 092 066 3
	OMP2	−76.265 470 576 8	0.724 729 427 6
	CC2	−76.265 519 413 7	0.716 397 141 7
NH_3	CCSD	−56.421 326 271 4	−0.575 380 861 1
	OMP2	−56.408 134 740 5	−0.570 946 397 5
	CC2	−56.408 016 476 4	−0.568 898 057 1
CH_4	CCSD	−40.394 143 335 9	0.0
	OMP2	−40.371 768 987 0	0.0
	CC2	−40.371 657 899 0	0.0

4 Comparison of absorption spectra from TDCC2 and LRCC2 theory

Figure 1 shows the agreement between spectra from TDCC2 simulations and from LRCC2 calculations using the Lanczos-chain-driven algorithm described by Coriani et al.[1,2] as implemented in the Dalton quantum chemistry program.[3,4] The same geometries and basis sets as above were used.

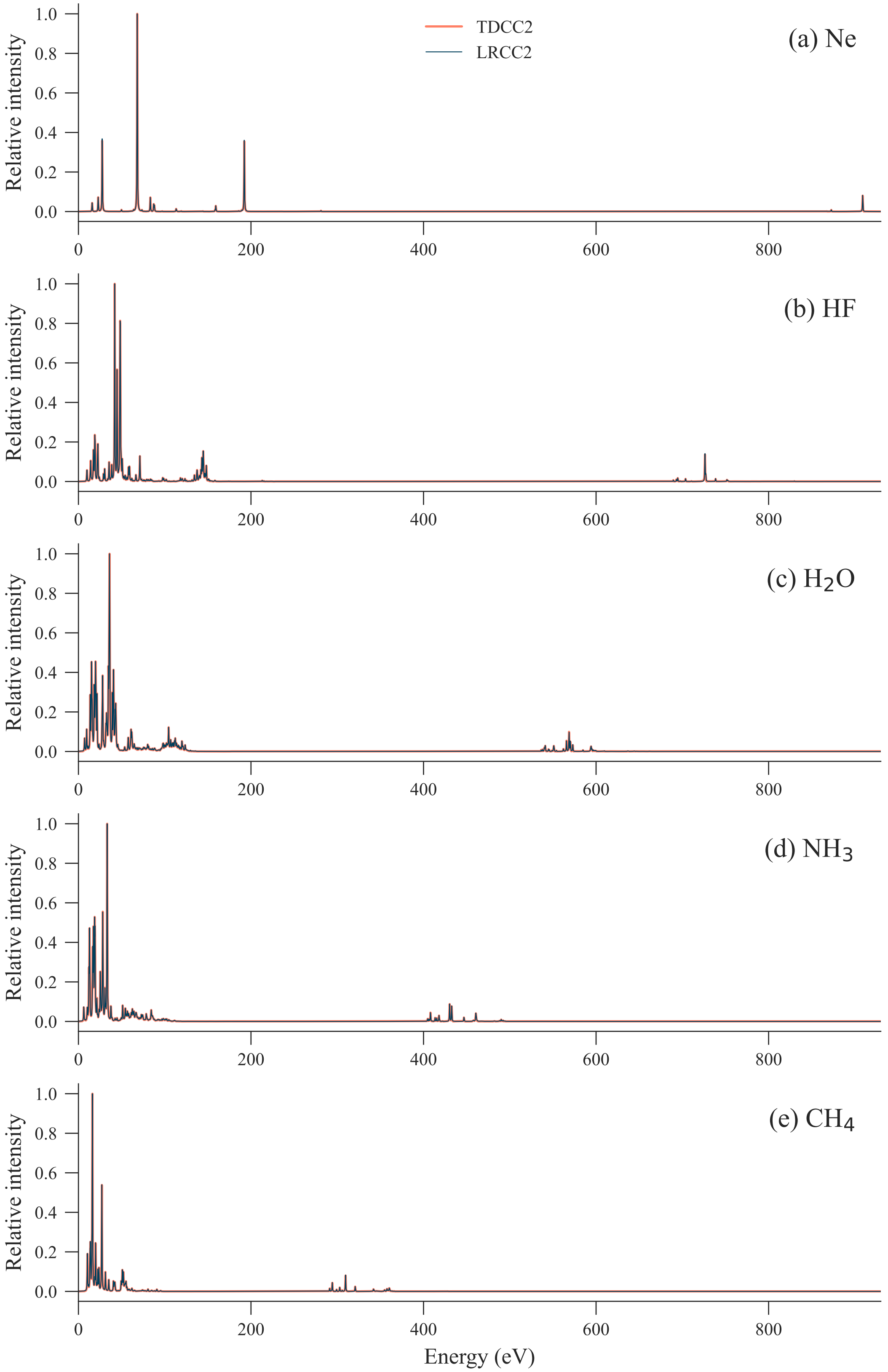

Figure 1: Absorption spectra from TDCC2 simulations and LRCC2 calculations for Ne, HF, H_2O, NH_3 and CH_4.

References

(1) Coriani, S.; Christiansen, O.; Fransson, T.; Norman, P. Coupled-cluster response theory for near-edge x-ray-absorption fine structure of atoms and molecules. *Phys. Rev. A* **2012**, *85*, 022507.

(2) Coriani, S.; Fransson, T.; Christiansen, O.; Norman, P. Asymmetric-Lanczos-Chain-Driven Implementation of Electronic Resonance Convergent Coupled-Cluster Linear Response Theory. *J. Chem. Theory Comput.* **2012**, *8*, 1616–1628.

(3) Aidas, K.; Angeli, C.; Bak, K. L.; Bakken, V.; Bast, R.; Boman, L.; Christiansen, O.; Cimiraglia, R.; Coriani, S.; Dahle, P.; Dalskov, E. K.; Ekström, U.; Enevoldsen, T.; Eriksen, J. J.; Ettenhuber, P.; Fernández, B.; Ferrighi, L.; Fliegl, H.; Frediani, L.; Hald, K.; Halkier, A.; Hättig, C.; Heiberg, H.; Helgaker, T.; Hennum, A. C.; Hettema, H.; Hjertenaes, E.; Høst, S.; Høyvik, I.-M.; Iozzi, M. F.; Jansík, B.; Jensen, H. J. A.; Jonsson, D.; Jørgensen, P.; Kauczor, J.; Kirpekar, S.; Kjaergaard, T.; Klopper, W.; Knecht, S.; Kobayashi, R.; Koch, H.; Kongsted, J.; Krapp, A.; Kristensen, K.; Ligabue, A.; Lutnaes, O. B.; Melo, J. I.; Mikkelsen, K. V.; Myhre, R. H.; Neiss, C.; Nielsen, C. B.; Norman, P.; Olsen, J.; Olsen, J. M. H.; Osted, A.; Packer, M. J.; Pawlowski, F.; Pedersen, T. B.; Provasi, P. F.; Reine, S.; Rinkevicius, Z.; Ruden, T. A.; Ruud, K.; Rybkin, V. V.; Sałek, P.; Samson, C. C. M.; Sánchez de Merás, A.; Saue, T.; Sauer, S. P. A.; Schimmelpfennig, B.; Sneskov, K.; Steindal, A. H.; Sylvester-Hvid, K. O.; Taylor, P. R.; Teale, A. M.; Tellgren, E. I.; Tew, D. P.; Thorvaldsen, A. J.; Thøgersen, L.; Vahtras, O.; Watson, M. A.; Wilson, D. J. D.; Ziolkowski, M.; Ågren, H. The Dalton quantum chemistry program system. *Wiley Interdiscip. Rev. Comput. Mol. Sci.* **2014**, *4*, 269–284.

(4) Olsen, J. M. H.; Reine, S.; Vahtras, O.; Kjellgren, E.; Reinholdt, P.; Hjorth Dundas, K. O.; Li, X.; Cukras, J.; Ringholm, M.; Hedegård, E. D.; Di Remigio, R.; List, N. H.; Faber, R.; Cabral Tenorio, B. N.; Bast, R.; Pedersen, T. B.; Rinkevicius, Z.; Sauer, S.

P. A.; Mikkelsen, K. V.; Kongsted, J.; Coriani, S.; Ruud, K.; Helgaker, T.; Jensen, H. J. A.; Norman, P. Dalton Project: A Python platform for molecular- and electronic-structure simulations of complex systems. *J. Chem. Phys.* **2020**, *152*, 214115.
